# Enhancement of rider comfort by magnetorheological elastomer based damping treatment at strategic locations of an electric two wheeler

**DOI:** 10.1038/s41598-024-70915-4

**Published:** 2024-08-29

**Authors:** Keerthan Krishna, G. T. Mahesha, Sriharsha Hegde, B. Satish Shenoy

**Affiliations:** Department of Aeronautical and Automobile Engineering, Manipal Institute of Technology, Manipal Academy of Higher Education, Manipal, Karnataka 576104 India

**Keywords:** Electric two-wheeler, Magnetorheological elastomer, Silicone rubber, Whole-body vibration, Rider’s comfort, Engineering, Mechanical engineering

## Abstract

The vibrations generated in the two-wheeled vehicles like motorcycles due to road irregularities such as cracks, potholes, and bumps on the road cause discomfort for the rider as well as the pillion. These vibrations are reported to cause lower back pains, musculoskeletal effects, fatigue, and long-term health issues. Particularly, electric two-wheelers are more susceptible to these vibrations caused by the road and need attention. This paper presents an innovative technique for the reduction of vibrations at prominent locations in the electric two-wheeler to improve the rider’s comfort. All measured accelerations are about vertical direction (along z-axis as per ISO 2631-1 standard). Passive and Semi-active damping treatments namely, Room temperature vulcanizing Silicone rubber and Magnetorheological elastomer (MRE) were applied on the test vehicle at strategic locations of vibration. Both were compared for their effectiveness in reducing the vibrations. Results showed that MRE based damping technique proved better vibration isolation at the strategic locations. The weighted root mean square acceleration as well as vibration dose values were found to decrease with the help of damping treatments thus improving the rider’s overall comfort level.

## Introduction

Whole-body vibration (WBV) experienced in vehicular environments, including automobiles such as trucks, buses, and two-wheeled and three-wheeled vehicles can give rise to a spectrum of adverse physiological effects on occupants. Prolonged exposure to mechanical vibrations during transportation is associated with increased risks of musculoskeletal discomfort, fatigue, and potential exacerbation of pre-existing conditions, particularly in the lumbar spine region^1–3^. Professional drivers, who spend extended durations in vehicular environments, are especially susceptible to occupational health concerns related to WBV. The continuous transmission of vibrations has implications for circulatory and nervous system function, potentially impacting cardiovascular health and contributing to heightened physiological stress^4,5^. Safety is a critical consideration, as excessive WBV may compromise a driver’s dynamic control over the vehicle-damping materials^6^. Ergonomic seating designs with optimal cushioning and support, as well as the integration of advanced vibration isolation systems, contribute to minimizing the transmission of vibrations to occupants^7^. Regulatory frameworks and standards may be instrumental in establishing acceptable levels of WBV in various vehicle types.

Two-wheelers, commonly referred to as motorcycles or scooters, have witnessed a surge in popularity owing to their affordability, convenience, and maneuverability^8–10^. From daily commuters to office employees, a diverse range of individuals rely on two-wheelers as their primary mode of transportation on a day-to-day basis. However, vibrations have long been a common issue for two-wheelers, causing discomfort, fatigue, and even potential safety hazards for riders^11–14^.

The vibrational challenges faced by two-wheelers, particularly electric two-wheelers are affected by the condition of the roads they traverse upon^15–18^. Electric two-wheelers, characterized by their lighter weight and stiffer suspensions in comparison to their gasoline-powered counterparts, are inherently more susceptible to vibrations induced by road imperfections^19^. The quality of roads plays a vital role in intensifying vibration-related problems. Poorly maintained roads, featuring potholes, cracks, and uneven surfaces, can intensify vibration issues in electric two-wheelers^20,21^. Such road defects contribute to abrupt changes in the vehicle’s suspension, leading to sudden jolts and vibrations that not only cause discomfort but also pose potential hazards to riders^22,23^.

The design of a two-wheeler emerges as a critical factor in mitigating the vibrations that can impact human health^24,25^. Specific locations on a two-wheeler, namely the handlebar, footrest, and rider’s seat, are of primary concern in the development of an effective design. Vibrations and shocks may transfer straight from the road to the rider through these locations, which are in constant contact with the human body. Although electric two-wheelers are appealing and cost-effective, they face difficulties from road vibrations and require careful maintenance^26,27^.

ISO 2631-1 is an international standard that provides guidelines for evaluating the effects of whole-body vibration (WBV) on human health, comfort, and perception. Developed by the International Organization for Standardization (ISO), this standard is critical for industries and environments where humans are exposed to mechanical vibrations, such as in vehicles, industrial machinery, and certain recreational activities. The primary aim of ISO 2631-1 is to ensure safety and well-being by establishing acceptable levels of vibration exposure and providing a framework for measuring and analyzing WBV^28^.

The standard specifies the use of frequency weighting to account for the varying sensitivity of the human body to different vibration frequencies. This weighting helps in more accurately assessing the potential health impacts of WBV. For instance, the human body is more sensitive to low-frequency vibrations (around 1–20 Hz), which can cause significant discomfort and health issues, especially in the lumbar spine region.

ISO 2631-1, the International Standard for evaluating human exposure to whole-body vibration, has been effectively employed in various contexts such as air, water, rail, and road transportation. This underscores its significance and reliability in assessing the impact of vibrations on human health and comfort across different modes of transport. In aviation, ISO 2631-1 is utilized to evaluate the vibrations experienced by passengers and crew members during flights. Aircraft, both commercial and military, are subject to various vibration sources such as turbulence, engine operations, and structural responses. By applying ISO 2631-1 standards, engineers and designers can assess the vibration levels and implement measures to minimize their impact, thereby enhancing passenger comfort and reducing potential health risks for the crew. This standard ensures that aircraft meet stringent vibration criteria, promoting safer and more comfortable air travel. For watercraft, including ships and boats, ISO 2631-1 provides a framework for measuring and assessing the vibrations transmitted to passengers and crew. Vibration sources on water vessels include engine operations, hull interactions with waves, and onboard machinery. Applying ISO 2631-1 helps in identifying critical vibration levels that may affect comfort and performance. This is particularly important for long voyages where prolonged exposure to vibrations can lead to fatigue and musculoskeletal issues. By adhering to this standard, the maritime industry can improve the design and operation of vessels to ensure better ride quality and health outcomes for those on board^29,30^.

In the rail industry, ISO 2631-1 is employed to evaluate the vibrations experienced by passengers and train operators. Rail vehicles are subject to vibrations from track irregularities, wheel–rail interactions, and onboard equipment. Using ISO 2631-1, engineers can measure vibration levels and assess their impact on comfort and health. This information is crucial for designing suspension systems, seating arrangements, and other components that mitigate vibrations. The application of this standard helps in enhancing passenger comfort, reducing operator fatigue, and improving the overall safety and efficiency of rail transport^31–33^.

For road vehicles, including cars, buses, and trucks, ISO 2631-1 is instrumental in assessing the vibrations transmitted to drivers and passengers. Road surface conditions, vehicle speed, and engine vibrations are some of the primary sources of whole-body vibration in road transport. By using ISO 2631-1, manufacturers can evaluate the vibration levels and make necessary design improvements to suspension systems, seating, and interior components. This standard ensures that vehicles provide a smoother and more comfortable ride, reducing the risk of vibration-related health issues such as lower back pain and fatigue^34,35^.

Recent years have seen impressive advances in technology in the two-wheeler industry, with a constant focus on enhancing rider comfort, safety, and overall experience. Semi-active damping systems (SADS) are one of the effective means of minimizing the oscillations caused due to road-related vibrations. These cutting-edge technologies have changed the dynamics of riding by giving riders higher ride comfort, stability, and control^36–38^.

Passive suspension systems, which offer a predefined level of damping based on fixed parameters, have historically been the norm for two-wheelers. Passive techniques, while somewhat successful, are unable to respond to the dynamic and unpredictable nature of varying road conditions and rider behavior^39^. This restriction may result in unfavorable riding conditions and affect riding stability, particularly in difficult terrain or adverse weather conditions.

Semi-active damping technologies have significantly influenced this situation since their development. These cutting-edge systems can dynamically modify the damping properties of the suspension in response to varying riding circumstances because they make use of cutting-edge sensors, actuators, and real-time control algorithms. Riders can travel with comfort and stability because of this adjustability, which makes the ride safer, more comfortable, and more pleasant^40^.

One of the efficient passive damping techniques is employing silicone rubber. They have high elasticity and are resistant to permanent loads. The ease of using silicone and its suitability to work with the widest temperature makes it more suitable for many applications^41^. The characteristics of silicone rubber to absorb vibrations have gained applications in motors, fans, and rotary machines^42^. The silicone rubber is prepared by blending a silicone elastomer base along with a curing agent^43–45^. Based on previous research works, silicone has proved to decrease the static and dynamic vibrations in machines^46^. Hence, silicone rubber is used in the current study as a damping treatment for the vibrations generated in the electric two-wheeler and to increase the rider’s comfort.

Magnetorheological elastomers (MRE) classified under the semi-active damping systems, change their properties with the application of an external magnetic field^47,48^. MRE is a group of viscoelastic materials prepared by a mixture of elastomers with magnetic particles. These magnetic particles alter the properties of the materials when external magnetic fields are applied. The properties such as stiffness and damping behavior of the material are altered under the application of an external magnetic field. MREs are used in various engineering applications such as adaptive damping systems, vibration control, and shape-changing applications^49^. Several factors, including viscoelasticity, particle friction, and hysteresis losses, lead to internal damping in MREs. When a material is exposed to dynamic stress or vibrations, internal damping causes mechanical energy to be dissipated inside the material. Internal damping improves the material's capacity to absorb energy and minimize vibrations, even if it may somewhat lessen the material's overall stiffness. MREs with internal damping are therefore useful for vibration isolation and damping applications as they can efficiently reduce vibrations and shock from being transmitted.

This paper focuses on rider comfort enhancement by presenting a comprehensive study on the application of silicone rubber and MRE-based damping treatments strategically employed at key locations on an electric two-wheeler. These strategic locations are those points in the vehicle where the rider’s body is in continuous contact throughout the ride. The study utilizes a retrofit Hero Honda CD 100 SS two-wheeler that has been transformed into an electric two-wheeler (E2W) in the laboratory. Various on-road tests have been conducted on this converted vehicle, simulating real-world riding conditions on the road, and the ensuing vibration accelerations have been carefully measured and analyzed. However, as there is no clear mention of safety levels of health in ISO 2631-1 standard, this study is focused on minimizing the vibrations acquired through testing^50^.

The methodology employed in this research is divided into three distinct parts, each catering to different aspects of the two-wheeler’s performance. First, testing is conducted at random speeds, incorporating sudden accelerations, decelerations, and braking to mimic a rider’s day-to-day riding modes. This phase aims to comprehensively assess the damping treatments’ efficacy in handling dynamic conditions. Subsequently, the study focuses on testing at a relatively slower speed of 20 kmph, focusing on the vehicle’s response under less intense vibrational scenarios. Finally, the research extends to a slightly higher speed of 30 kmph, acquiring accelerations to study the potential risks associated with increased vibrations. These different speeds are considered to observe the response of the system at different riding styles of the ride. Throughout these tests, bituminous asphalt roads are chosen as the testing grounds, mirroring real-world conditions and particularly an Indian road scenario. National Instrument’s (NI) data acquisition systems and LabVIEW 2016 software were instrumental in facilitating data acquisition, with strategically placed Piezotronics accelerometers capturing vibration signals in adherence to ISO 2631-1 standard^28^.

Summarily, this paper identifies the challenges posed by vibrations in electric two-wheelers and provides a systematic approach to address these issues through the application of damping treatments. By employing on-road testing methodology utilizing data acquisition technologies, the study aims to contribute to the design and development of electric two-wheelers, enhancing rider comfort and safety.

## Methodology

### Test track

**Fig. 1:**
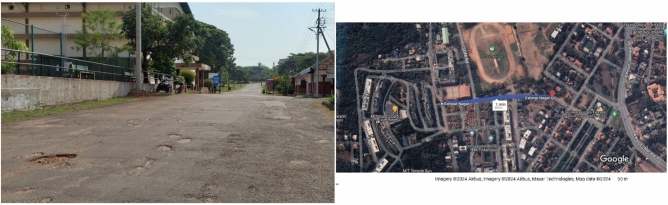
Testing track with potholes and humps^51^.

Bituminous asphalt road (Latitude: 13.3446830, Longitude: 74.7924247) with irregularities such as cracks, potholes, and humps are considered for the testing as shown in Fig. 1. All experiments are conducted as per the guidelines and regulations in ISO 2631-1 standard. The testing track is as shown in the Fig. 1.

### Test vehicle

**Fig. 2:**
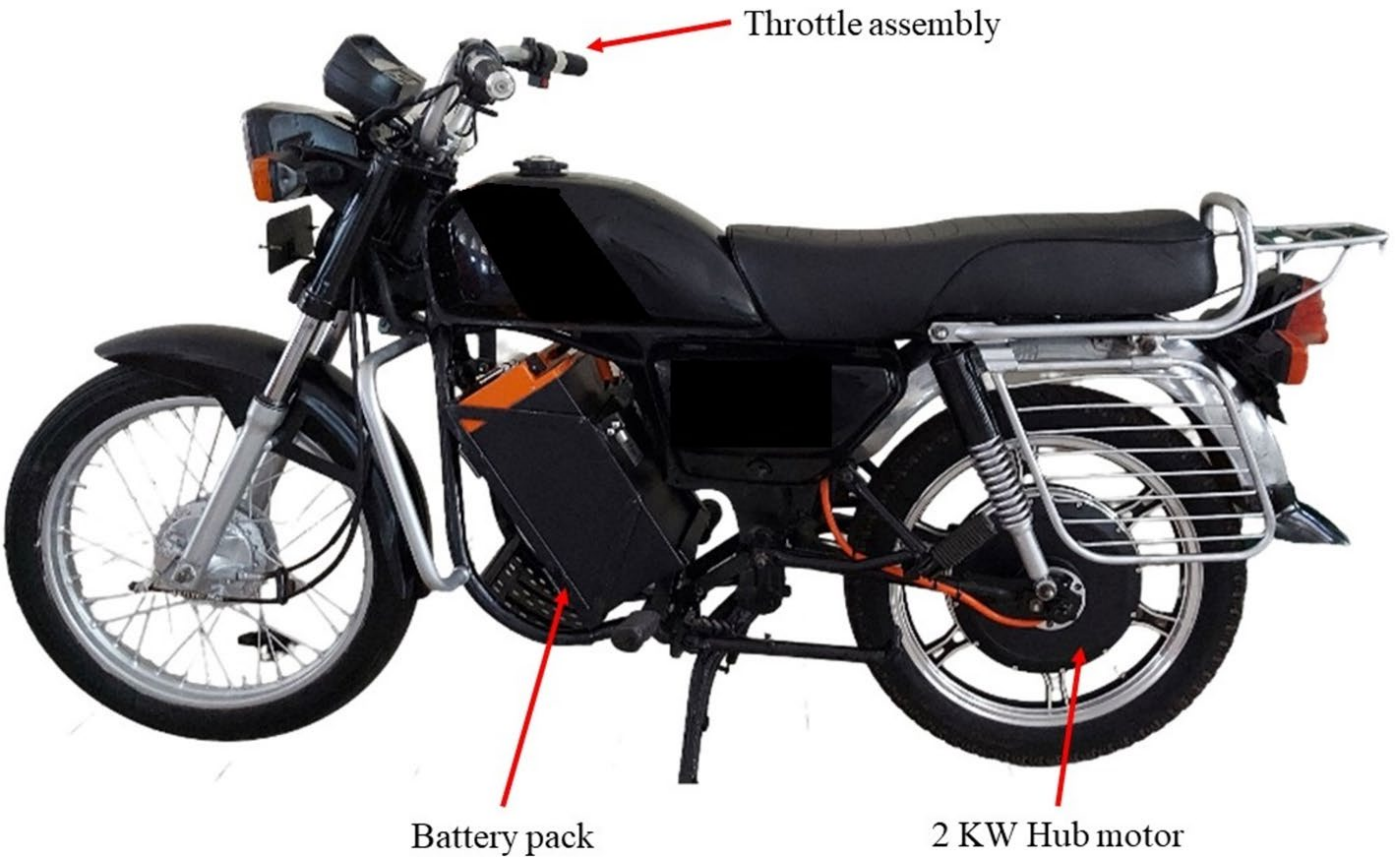
Electric two-wheeler (E2W).

The vehicle considered Hero Honda CD 100 SS was successfully converted to E2W at the laboratory and all the tests were carried out on the same vehicle. The overall weight including the weight of the vehicle (104.26 kg), the weight of the rider (80 kg), and pillion (85 kg) was about 269 kg. The handlebar, footrest, and seat were considered as the strategic locations of vibrations as these points in the vehicle would be in continuous contact with the human body. These locations were chosen as strategic points based on a comprehensive analysis of human–vehicle interaction, vibration pathways, and the impact on rider comfort and health. The handlebar, footrest, and seat are direct transmission paths for vibrations, making them critical for assessing vibration effects. Empirical studies and industry standards in automotive and ergonomics fields emphasize these points for vibration analysis and mitigation. Accelerometers were mounted at these strategic locations to acquire vibration amplitudes while riding. The laboratory built E2W is shown in Fig. 2 which indicates the main components of the system.

### Fabrication of damping material

RTV silicone rubber from Dow Corning Corporation, India (Silastic 3483 base and Silastic 83 curing agent) was used in the fabrication of silicone rubber. It involved mixing silicone gel with the hardener and curing at room temperature. The silicone gel with a density of 1100 kg/m^3^ was mixed with 3% hardener (as per supplier’s recommendations) taking the suitable amount of gel. The mixture was then poured into an acrylic mold and allowed to cure for 24 h.

**Fig. 3:**
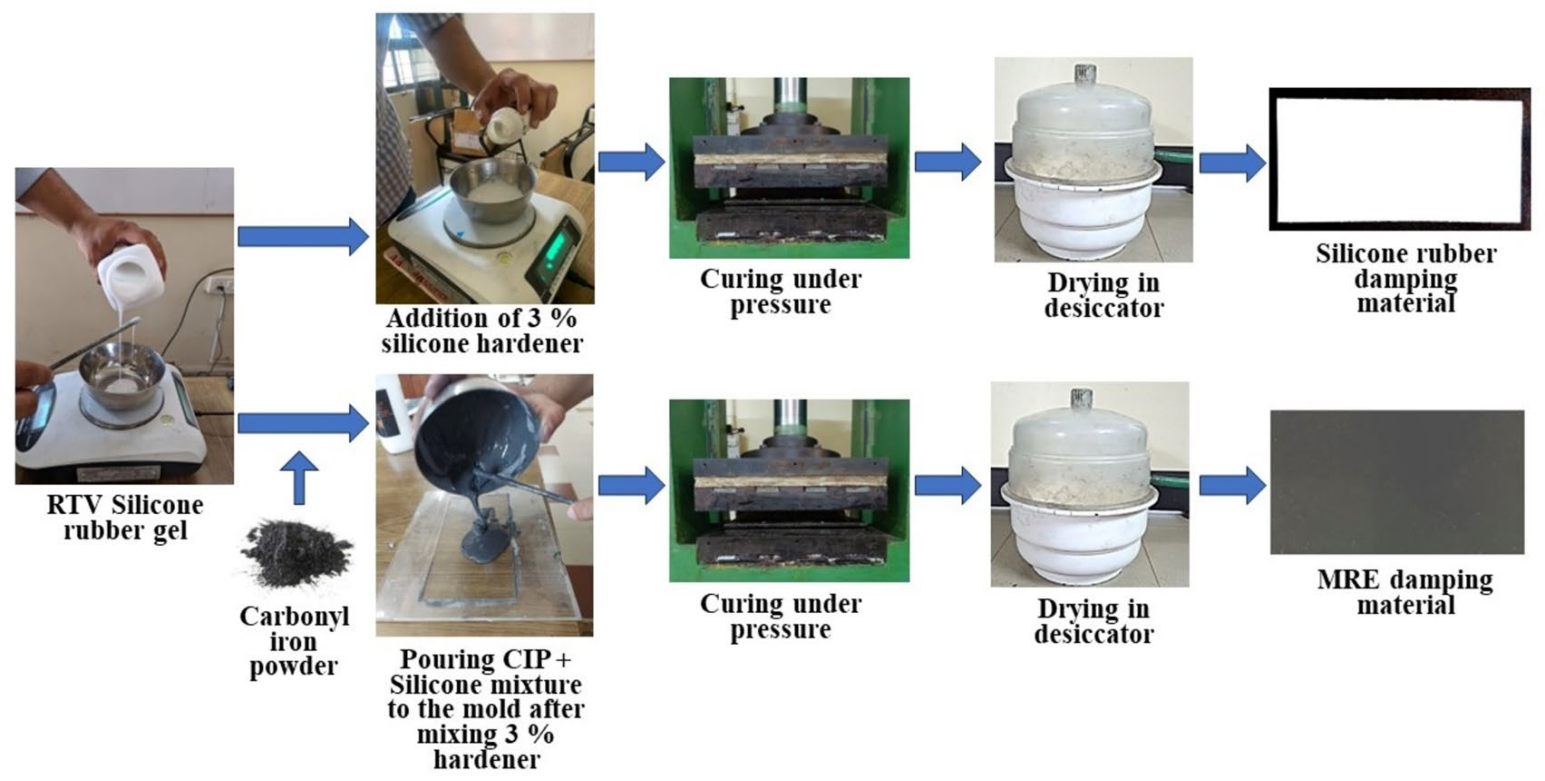
Preparation of the damping material.

Fabrication of MRE involved mixing the RTV silicone and hardener with Carbonyl Iron powder (CIP). An average particle size of 3.36 µ of CIP (Chengdu Nuclear 857 New Materials Ltd, China) was used in the current work. Based on the literature review a ratio of 75:25 mixture is found to be the optimum mixture for the preparation of MRE^52,53^. The mixture was added with 3% hardener (100:3) as per the suggestions of the manufacturer similar to preparation of silicone rubber. The mixture was kept inside a vacuum chamber to remove air bubbles. It is further cured at room temperature and cut to suitable sizes as applicable at the strategic locations of vibration on the E2W. The fabrication process of Silicone and MRE and the formed final material is shown in Fig. 3.

### Tensile test

**Fig. 4:**
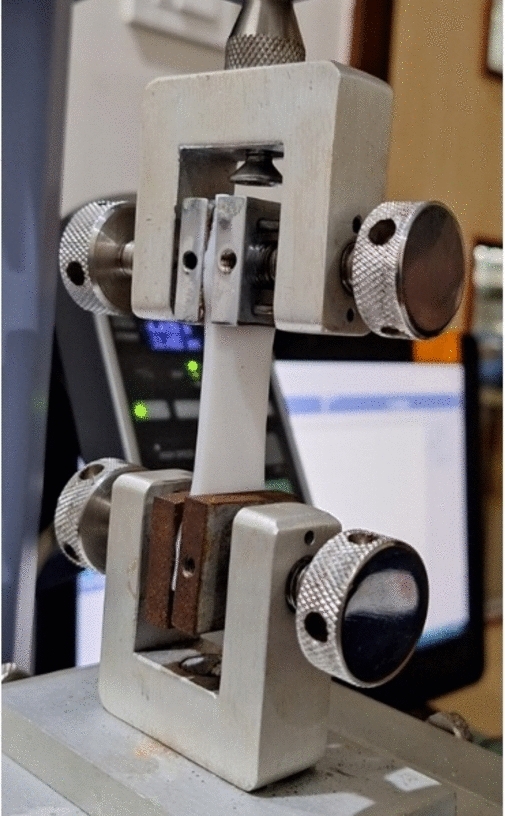
Tensile testing of silicone rubber.

**Fig. 5:**
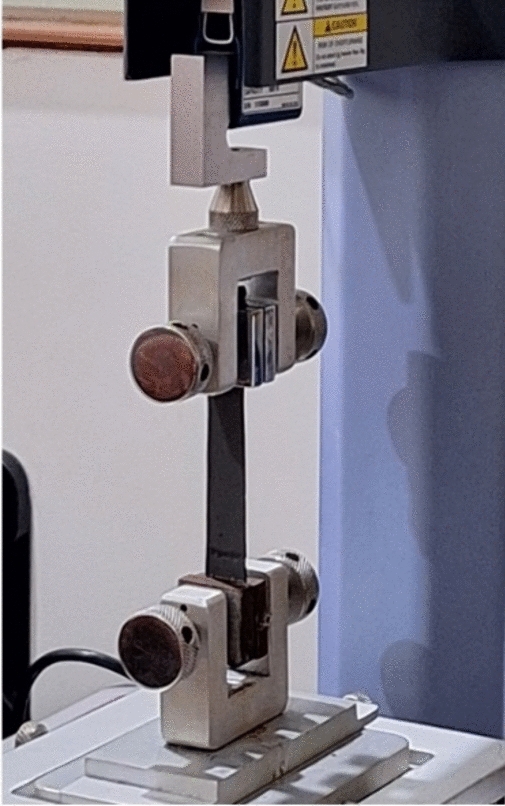
Tensile testing of MRE.

A universal testing machine from Shimadzu with a maximum load capacity of 500 N was used to conduct tensile tests for both Silicone rubber and MRE damping materials. The test specimen's gauge length (34 mm) was subjected to tensile testing at room temperature as per ASTM D412 standard. The samples are shown in Figs. 4 and 5.

### Procedure

The strategic locations of vibrations in the two-wheeler, namely the handlebar, footrest, and seat were considered for the test as these are prominent regions where the human body is in contact^54,55^. The study was conducted considering an average Indian road condition considering potholes and irregular humps. The testing speeds considered mimic typical city driving conditions at peak hours where more than 30 kmph is hard to achieve. In this regard, three driving cycles considered for testing were the random speed test (which is a combination of different speeds driven randomly, not more than 30 kmph) for about 1 km distance, a slow speed (20 kmph) for ~ 300 m, and a slightly higher speed (30 kmph) for ~ 300 m. The testing track considered was almost a no-traffic zone, inside the campus where humps and potholes were present. The speed was controlled manually, and it was kept constant for 20 kmph and 30 kmph tests. Due to near zero traffic conditions, controlling the speed was achieved to maximum possible accuracy. Each test condition was repeated three times and averaged in order to ensure the repeatability of values. The test was initially conducted without any damping treatment and later tested with silicone rubber and MRE (with and without magnetic field). The vibrations generated at the strategic locations in the vertical direction (z-axis as per ISO 2631-1) were captured using NI’s data acquisition system and LabVIEW 2016 software. The accelerometers used were PCB Piezotronics made of sensitivity 101.6, 100.7, 103.6, and 101.2 mV/g respectively at the left and right side of the handlebar, footrest, and seat. To gather the findings from all the places simultaneously, accelerometers are installed at each strategic location. Here, every test is run three times, following the same path and the average of the results is used to determine the outcome. In all the cases the magnetic field for MRE had been set up using permanent neodymium magnets. A magnetic field of 0.0075 T was obtained at the center of the damping material as checked by an SES Instruments-made digital Gauss meter.

### Handlebar

**Fig. 6:**
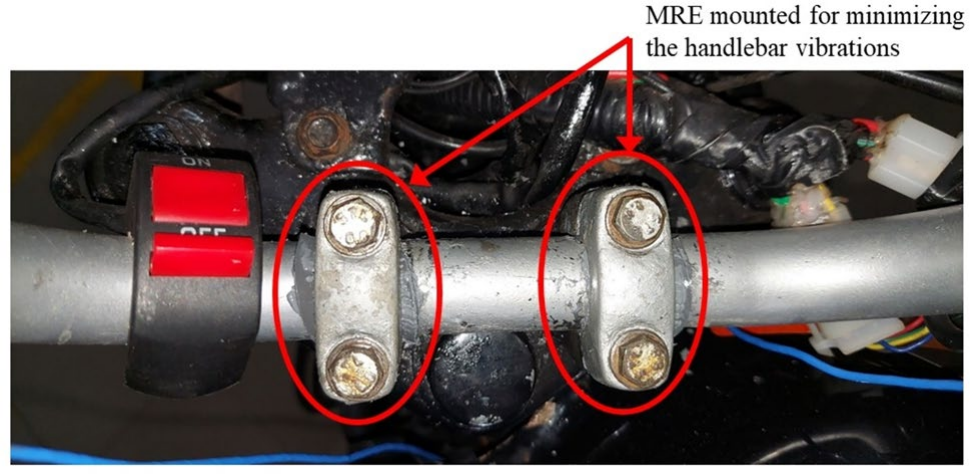
MRE damping material mounted at the handlebar.

The damping treatment at the handlebar was mounted at the vibration source which is the handlebar mount. Accelerometers were placed at both the left and right side of the handlebar and were mounted as per guidelines of ISO 2631-1. Damping material was embedded in the handlebar mount because it is the fixing point of the left and right sides of the handlebar. Figure 6 shows the position of the mounted damping material (MRE is shown as a sample here).

### Footrest

**Fig. 7:**
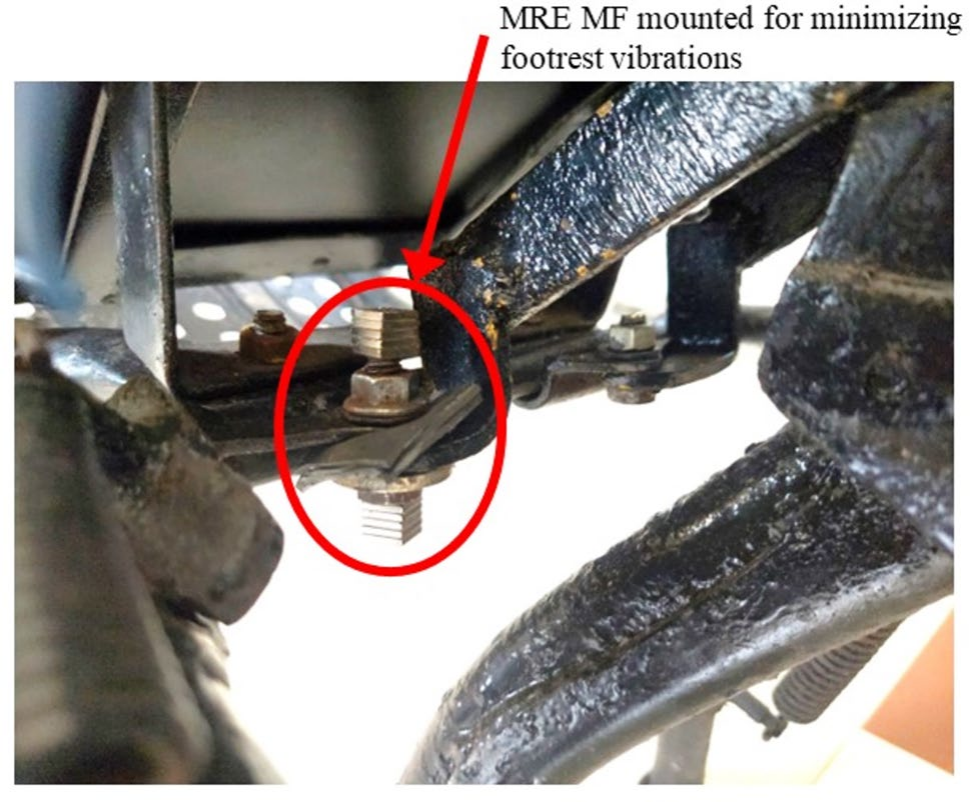
MRE MF damping material mounted for the footrest.

The damping treatment for the footrest was inculcated at the footrest mount as shown in Fig. 7. The accelerometer was mounted near the place where the foot is often placed (as per ISO 2631-1 standard). Figure 7 shows the position of the mounted damping material (MRE MF is shown as a sample here). The E2W footrest which is connected to the frame of the vehicle is a prominent point that transfers vibration to the human body.

### Seat

**Fig. 8:**
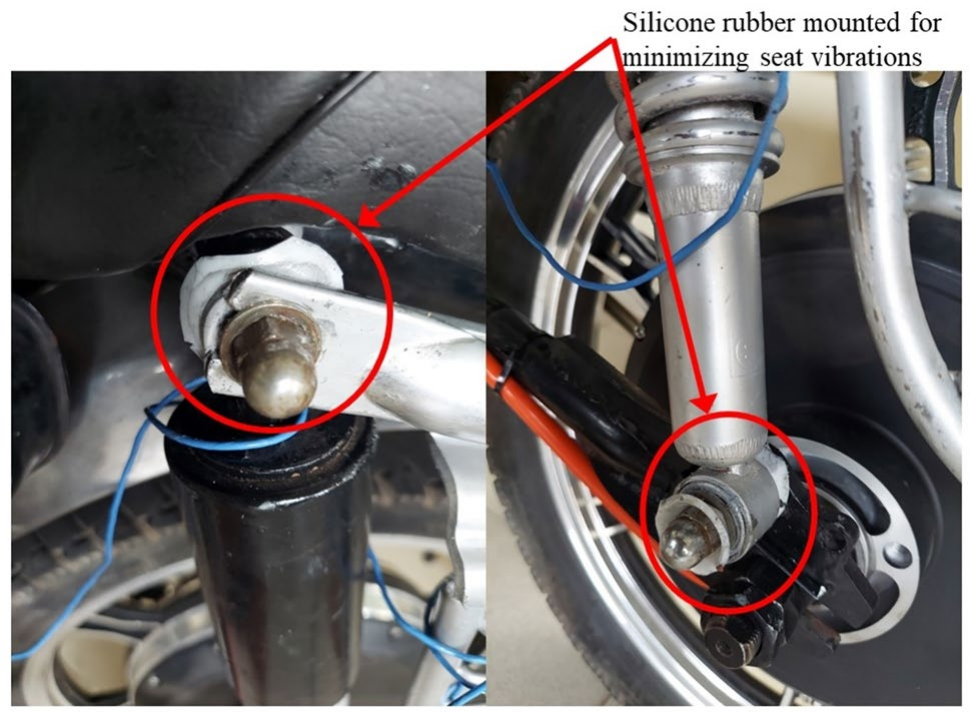
Silicone rubber damping material mounted for seat.

The rear shock absorber of the test vehicle viz. endurance rear shocker was selected for the damping treatment. The damping materials viz. silicone rubber, MRE, and MRE MF were accommodated at both the top and bottom of the shocker to maintain uniformity. The mounting position of the damping material (silicone rubber is shown as a sample here) is shown in Fig. 8.

## Analysis

National instrument’s data acquisition systems (cDAQ-9191 and NI-9234) were used to acquire the vibration signals^56^. Tom Irvine’s^57^ MATLAB GUI multi-function signal analysis package was used to calculate the weighted acceleration and VDV. Here, ‘*W*_*k*_’ weighting factor is used to calculate the VDV as ‘*W*_*k*_’ is specifically designed to evaluate whole-body vibration in the vertical direction (z-axis), which is most relevant for riders of two-wheelers due to the predominant vibration direction experienced while riding. Equations (1), (2) and (3) show the formulas to calculate the weighted RMS acceleration, VDV as per ISO 2631-1 standard.

*W*_*k*_ is the frequency weighting factor specified for evaluating whole-body vibration exposure in the vertical (z-axis) direction when a person is seated or standing on a vibrating surface as per ISO 2631-1 standard^28^. This weighting is particularly relevant in assessing vibrations that occur in vehicles, industrial machinery, and other environments where vertical vibrations are predominant.

The *W*_*k*_ weighting curve accounts for the fact that the human body is more sensitive to certain frequencies in the vertical direction. Typically, frequencies around 1–2 Hz are most critical, as they correspond to the natural frequencies of the human body and can cause significant discomfort or health issues. The *W*_*k*_ weighting attenuates higher and lower frequencies to reflect this sensitivity accurately.

In this research on vibration analysis, particularly focused on the vibration analysis and optimization of the handlebar, footrest, and seat of a two-wheeler, the application of the *W*_*k*_ weighting factor is crucial. The handlebar, footrest, and seat are the primary contact points between the rider and the vehicle, and vertical vibrations transmitted through these points can significantly affect rider comfort and control. By applying the *W*_*k*_ weighting factor to the vertical vibrations experienced at these contact points, the impact of specific vibration frequencies on the rider’s hands, foot, and lower back can be accessed.

1 $$ {\text{RMS }}\left( {{\text{m}}/{\text{s}}^{2} } \right) = \left[ {1/{\text{T}}\mathop {\mathop \smallint \limits^{{\text{T}}} }\limits_{0} {\text{a}}^{2} \left( {\text{t}} \right){\text{dt}}} \right]^{{1/2}} , $$

2 $$ {\text{VDV }}\left( {{\text{m}}/{\text{s}}^{{1.75}} } \right) = \left( {\mathop {\mathop \smallint \limits^{{\text{T}}} }\limits_{0} {\text{a}}^{4} \left( {\text{t}} \right){\text{dt}}} \right)^{{1/4}} , $$

3 $${a}_{w}= {\left[\sum_{i}{\left({W}_{i}{a}_{i}\right)}^{2}\right]}^{1/2},$$

where *a*(*t*) is the frequency-weighted acceleration time history. *T* is the duration of measurements in seconds. W_i_ is the weighting factor as given by ISO 2631-1^28^.

## Results and discussion

### Stress–strain behavior

**Fig. 9:**
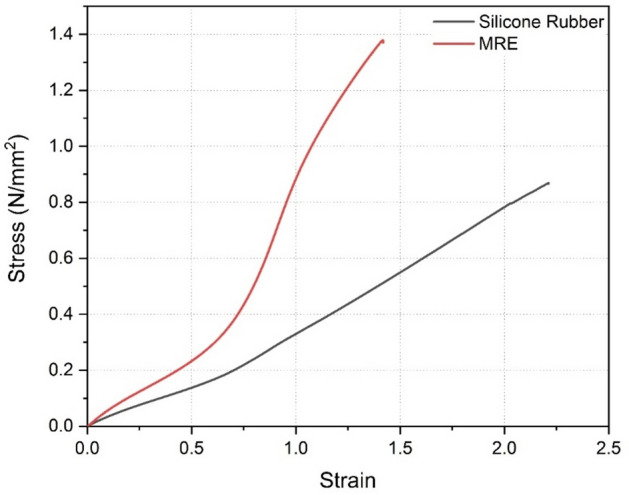
Stress–strain behaviour of samples.

Figure 9 shows the stress–strain behavior of the MRE, and silicone rubber samples tested at room temperature. From the stress–strain graph, it was observed that the stiffness modulus of MRE is higher than that of silicone rubber. Similarly, silicone rubber exhibited higher ductility than MRE. This is due to the reason that MRE contains CIP particles embedded in them and these increase the stiffness of the MRE sample. It was also noted that, with an increase in CIP percentage in the matrix the stiffness increased as well^58^. From Fig. 9 the stiffness modulus of silicone rubber was found to be 0.45 MPa and of MRE was 1.02 MPa in the absence of magnetic field.

### Study 1 (random speed test)

**Fig. 10:**
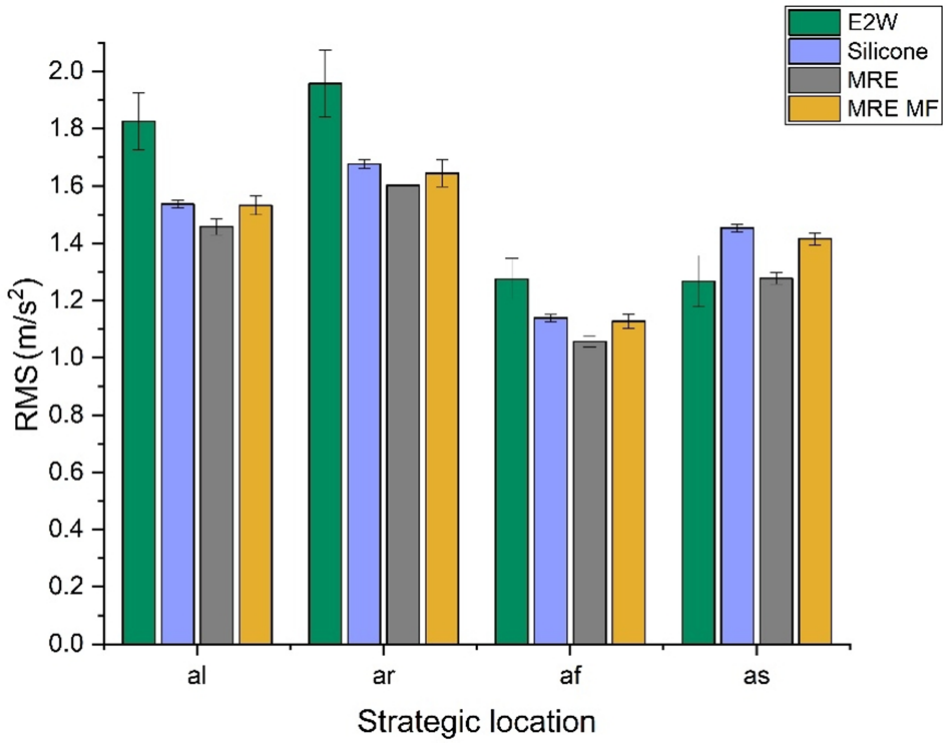
Weighted RMS comparison at random speed.

**Fig. 11:**
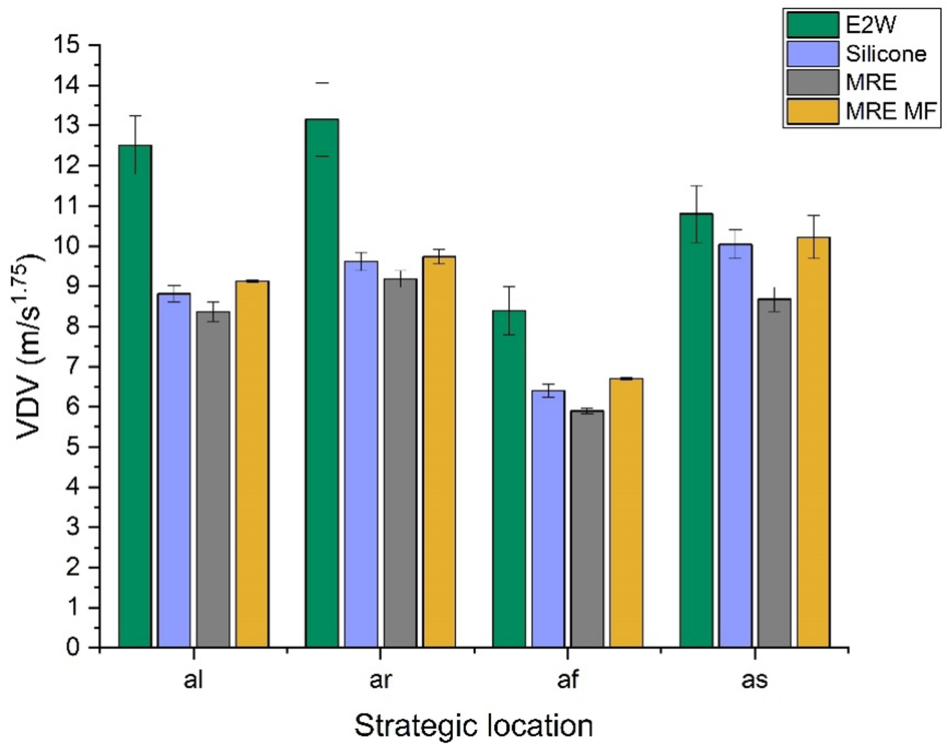
VDV comparison at random speed.

The comparison of whole-body vibrations (WBV) in the case of original E2W and E2W treated with silicone rubber, MRE, and MRE with the magnetic field (MRE MF) at different speed tests is shown in studies 1, 2, and 3. The first study refers to the vehicle driven at random speeds. Figures 10 and 11 show the weighted RMS acceleration and VDV at the left side of the handlebar (*al*), right side of the handlebar (*ar*), footrest (*af*), and seat (*as*) respectively. In the graphs illustrating RMS acceleration and VDV, error bars have been included to represent the standard deviation, thereby showing the variability and repeatability of the measurements across different trials.

From the random speed test, it was seen that the vibration levels at all the strategic locations were decreased with the application of damping materials. MRE treatment proved to be a better vibration isolator compared to silicone and MRE with the magnetic field by reducing 20.16, 18.13, 17.17, and − 0.78% of weighted RMS acceleration as shown in Fig. 10 when compared with E2W without any damping treatment. Further analysis as shown in Fig. 11 shows the decrement in the VDV by 33.13, 30.20, 29.75, and 19.67% at *al*,* ar, af,* and *as* respectively. This showed the effectiveness of the MRE treatment at strategic locations in the reduction of vibrations. Here, MRE with magnetic field showed a decrease in VDV as well as weighted acceleration. However, MRE without the magnetic field showed a further decrease in values and was better suited at random speed. This is due to the material’s inherent nonlinear damping properties, which enable it to respond more dynamically and effectively to the wide range of vibrations encountered during random speed tests. Unlike the magnetic field-enhanced MRE, which may have a more specific optimized range of effectiveness, the standard MRE can adapt more flexibly to the constantly changing vibration patterns, thereby reducing vibrations more effectively under random speed conditions^59^.

### Study 2 (20 kmph speed test)

**Fig. 12:**
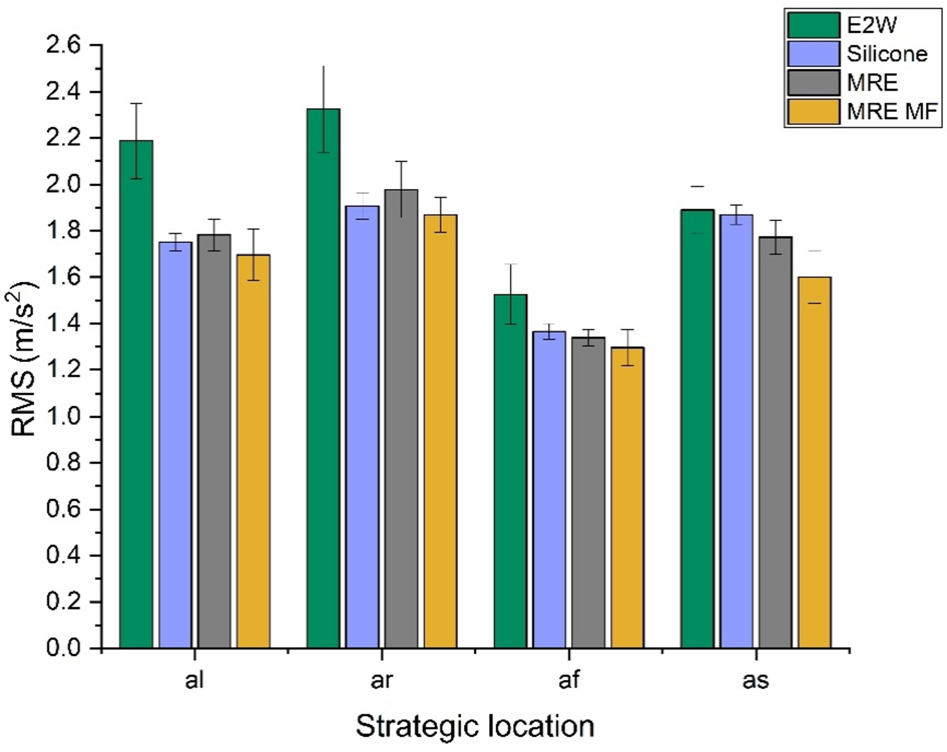
Weighted RMS comparison at 20 kmph.

**Fig. 13:**
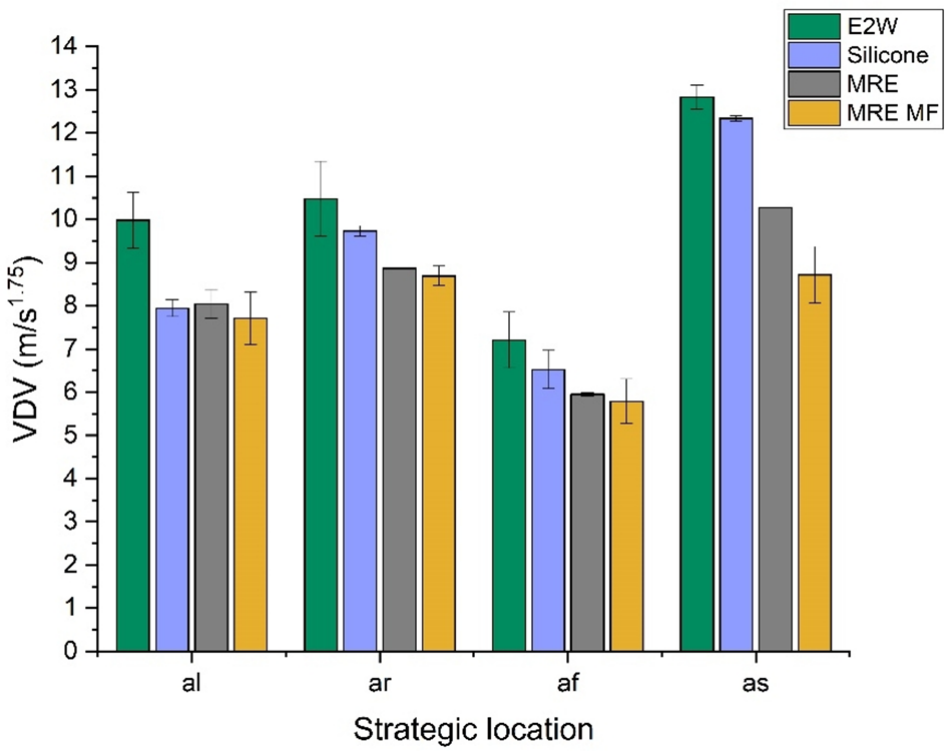
VDV comparison at 20 kmph.

Study 2 shows the comparison of the WBV with the two-wheeler at 20 kmph speed test. Figures 12 and 13 show that MRE MF treatment at the strategic locations showed better results compared to silicone and MRE. The weighted RMS acceleration at the strategic locations in Fig. 12 indicates a decrease of 22.50, 19.64, 15.06, and 15.42%, and VDV in Fig. 13 shows that, about 22.74, 17.04, 19.68, and 32.05% of decrement in vibrations in VDV at *al*,* ar, af* and *as* respectively. When a magnetic field is applied to an MRE, the embedded magnetic particles align themselves with the direction of the magnetic field. This alignment enhances the interparticle interactions, leading to an increase in the stiffness of the material. Consequently, the storage modulus (G′) of the MRE increases with the application of the magnetic field. This increase in stiffness allows MREs to exhibit controllable mechanical properties, making them suitable for various applications such as vibration control and adaptive structures.

### Study 3 (30 kmph speed test)

**Fig. 14:**
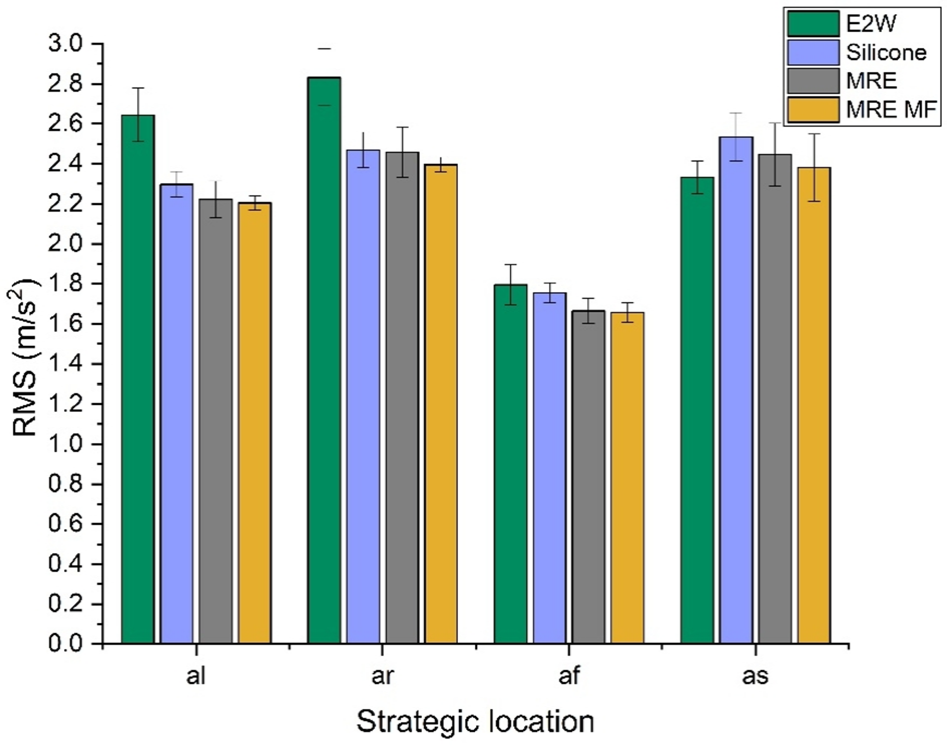
Weighted RMS comparison at 30 kmph.

**Fig. 15:**
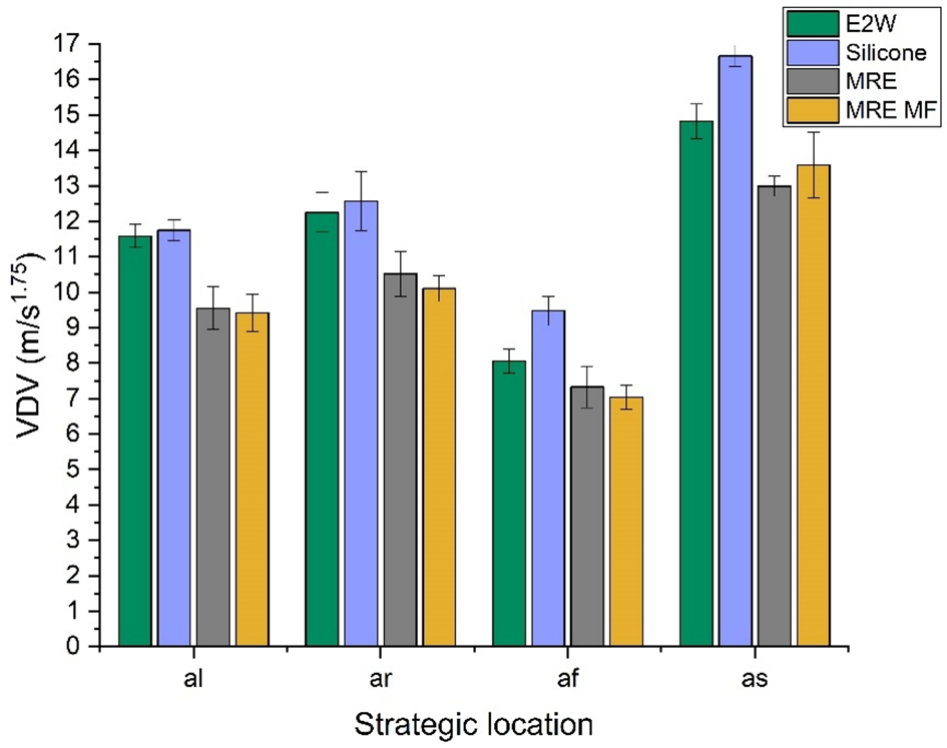
VDV comparison at 30 kmph.

Study 3 shows the comparison of vibrations at a slightly higher speed of 30 kmph. The weighted RMS acceleration in Fig. 14 shows about 16.62, 15.42, 7.62, and − 2.04% of the decrease in weighted acceleration, and VDV in Fig. 15 shows 18.69, 17.49, 12.67, and 8.30% of the decrease in VDV at the strategic locations *al, ar, af* and *as* respectively by using MRE MF.

In all the above cases, the damping treatments were effective in reducing the vibrations generated due to road irregularities in the electric two-wheeler. Silicone rubber, even though it reduced vibrations in some cases was not completely reliable as the damping was not effective at some speeds as seen in Fig. 15. The treatment of MRE with a magnetic field shows excellent vibration damping than MRE without the magnetic field in most cases. This behavior of MRE when a magnetic field is applied results from an increase in the material’s storage modulus. The material becomes stiffer in the presence of a magnetic field, which causes it to store energy elastically. This effect occurs because of the particle alignment in the MRE under the influence of a magnetic field forming chain-like structures inside the material increasing the material;s internal stiffness and hence increasing its ability to dissipate vibrations.

### Statistical analysis of results

**Fig. 16:**
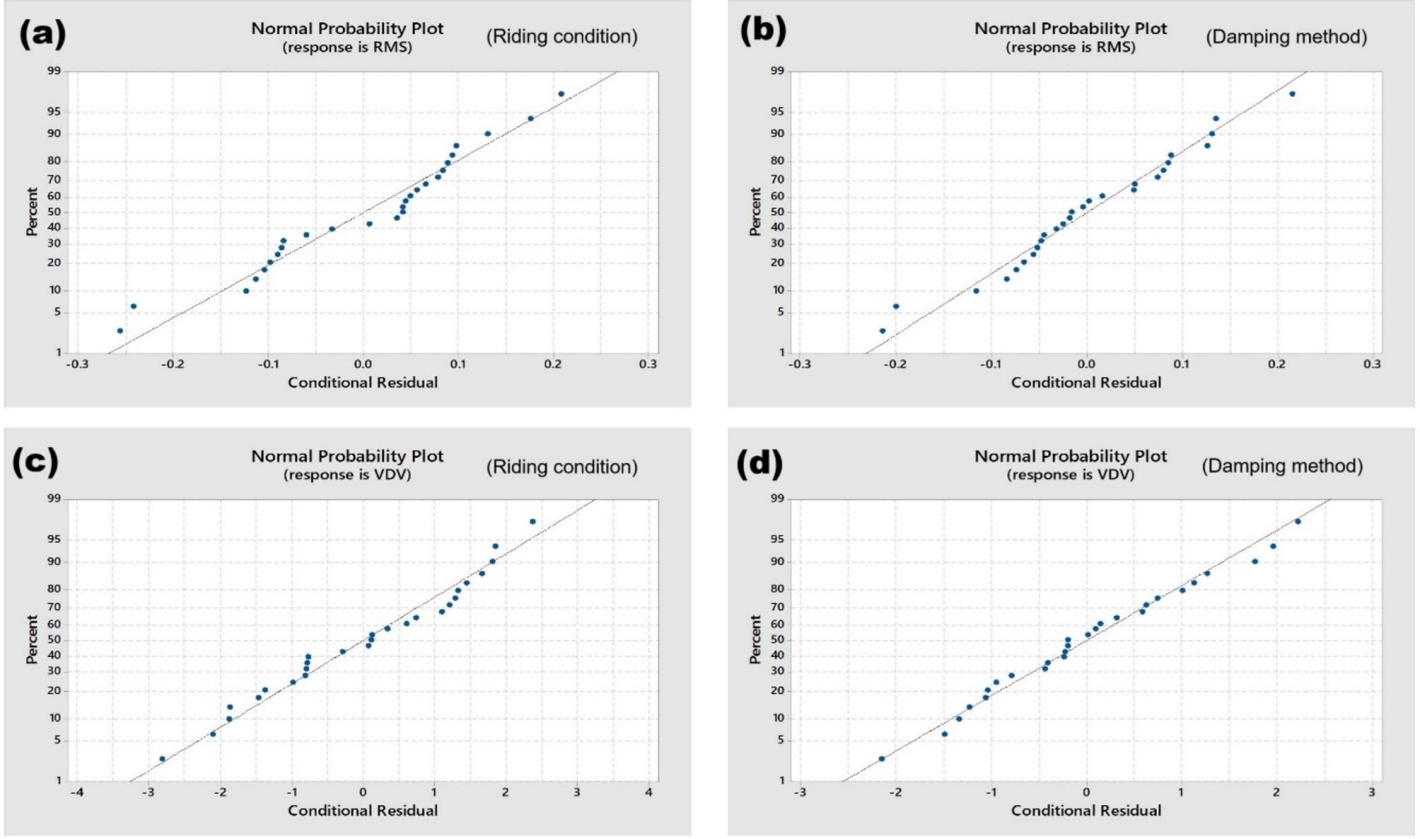
Norm plot of conditional residuals of response RMS for (**a**) riding condition, and (**b**) damping method, response VDV for (**c**) riding condition, and (**d**) damping method.

According to the results of ANOVA, it is observed from Table 1 that both the Riding condition and damping methods are significant, and hence, it can be concluded that both factors influence the values of weighted acceleration and VDV across different trials. Figure 16 shows the normal probability plots, each depicting the residuals of a response variable against a theoretical normal distribution.

**Table 1:** Mixed effects ANOVA model results comparing trial values across different variables.

Dependent variable	Independent variable	P value
Weighted RMS Acceleration (m/s^2^)	Riding condition	0.027
	Damping method	0.037
Vibration dose value (m/s^1.75^)	Riding condition	0.016
	Damping method	0.005

In the plots for RMS (a & b) and VDV (c & d) the residuals under both the Riding Condition and Damping Method contexts closely follow the theoretical normal distribution line, with minor deviations at the trials. This suggests that the residuals are approximately normally distributed, across different trials.

## Conclusion

This research investigates three methods of damping treatment incorporated at the strategic locations of an electric two-wheeler to reduce the vibrations generated due to road irregularities. The experimental method includes testing the two-wheeler on an actual road considering both the rider as well as the pillion. Three different conditions of tests were considered i.e., random speed, 20 kmph, and 30 kmph tests. The responses were measured at three strategic locations of the E2W test vehicle i.e., handlebar, footrest, and seat. From the study the following conclusions are drawn:

- The application of a magnetic field typically increases the storage modulus of an MRE by aligning the embedded magnetic particles, thereby enhancing the material’s stiffness. Meanwhile, internal damping within the MRE contributes to its ability to dissipate mechanical energy and damp vibrations, improving its vibration control capabilities.
- At random speed test, MRE based damping technique proved better in damping vibrations than silicone rubber and MRE with magnetic field. The MRE method reduced about 13.67% of weighted RMS acceleration and 28.19% of vibration dose value when compared to E2W with no damping treatment, considering the average of all the strategic locations.
- At the 20 kmph speed test, the treatment of MRE with a magnetic field at the strategic locations reduced about 18.15% of weighted acceleration and about 22.88% of VDV when compared to E2W without any damping treatments.
- At the 30 kmph speed test, the treatment of MRE with a magnetic field showed consistency in reducing vibrations by reducing about 9.40% of weighted acceleration and about 14.29% of VDV in the E2W.
- From the statistical analysis of different trials, it is observed that both the riding condition as well as damping methods are significant in assessing the RMS acceleration and VDV.
- From the overall study, it depicts that MRE based damping treatments at different strategic locations i.e., handlebar, footrest, and seat proved to reduce the vibrations and hence enhance the rider’s comfort. In the current testing phase of this study, permanent magnets were utilized to investigate the rheological properties of Magnetorheological Elastomers (MRE). This approach was chosen to provide a consistent and controllable magnetic field, enabling us to systematically analyze how the MRE behaves under magnetic influence. Permanent magnets offer a straightforward and cost-effective means to apply a magnetic field without the need for complex control systems. However, by identifying more strategic locations for the two-wheeler and incorporating an electromagnet where the magnetic field can be controlled by adjusting the input current, the damping properties can be regulated. This represents the future scope of this study.

## Data Availability

The datasets generated during and/or analyzed during the current study are available from the corresponding author upon reasonable request.

## Abbreviations

*af*: Weighted acceleration at footrest
*al*: Weighted acceleration at left side of handlebar
*ar*: Weighted acceleration at right side of the handlebar
*as*: Weighted acceleration at seat
CIP: Carbonyl iron particle
E2W: Electric two-wheeler
ISO: International Organization for Standardization
MRE: Magnetorheological elastomer
MRE MF: Magnetorheological elastomer with magnetic field
RMS: Root mean square
RTV: Room temperature vulcanizing
SADS: Semi-active damping systems
SR: Silicone rubber
VDV: Vibration dose value
WBV: Whole-body vibration
